# Patient Attitudes and Perceived Barriers Toward Mental Health Treatment Options in a Rural Student-Run Clinic

**DOI:** 10.7759/cureus.50667

**Published:** 2023-12-17

**Authors:** Maren L Downing, Mariah Hydzik, Godwin Y Dogbey, Thomas Motyka

**Affiliations:** 1 School of Osteopathic Medicine, Campbell University, Lillington, USA

**Keywords:** psycho-behavioral, hispanic population, rural setting, rural america, rural psychiatry, perceived barriers, osteopathic manipulative medicine, student run free clinic, mental health treatment

## Abstract

Background

While the prevalence of mental health conditions is similar in rural areas and non-rural areas, access to mental health care is more limited in rural areas. Patient attitudes toward specific mental health treatment options in rural populations have been understudied. Some previous studies indicate potential positive outcomes using osteopathic manipulative treatment (OMT) as an adjunct for mental health care. Physicians using OMT are more heavily represented in rural areas. Hence, understanding the mental health treatment needs and option preferences of the rural could inform policies that increase underserved population's access to various mental health treatment modalities including OMT. This study aims to characterize patient attitudes toward, beliefs regarding, and perceived barriers to treatment options for mental health treatment, access, and care in a rural, underserved clinical setting.

Methods

Adult patients attending a single outpatient rural clinic over a four-month period in 2022 were screened for participation. The survey consisted of Likert scale graded questions about mental health treatment options, access, knowledge, and perceived barriers including qualitative questions about OMTs. Versions of the survey were created in English and Spanish languages.

Results

Out of 46 respondents, 25 were English-speaking and 21 were Spanish-speaking. The most popular mental health treatments by respondents were indicated as therapy, spiritual guidance, and modifying diet and exercise. Considering barriers to care, 61% of respondents indicated cost of treatment as a logistical barrier. Finally, 80.5% of respondents did not have a good understanding of OMT.

Conclusions

The knowledge and understanding of patients’ perceived attitudes and barriers toward mental health care, inclusive of OMT, can provide insight to clinicians to improve patient outcomes and guide efforts in overcoming barriers to increase and expand mental health treatment availability and utilization by patients.

## Introduction

According to the United States (U.S.) Census Bureau, one in five Americans live in rural geographic areas. A 2016 Centers for Disease Control and Prevention report indicated that those living in rural areas are five times more likely to die from common preventable diseases compared to those living in urban areas [1]. The prevalence of mental health conditions is similar in rural areas to non-rural areas, but patients in rural areas are less likely to access care and receive treatment as their non-rural counterparts [2]. In addition, the rural patient population often presents later in their disease course due to poor access to healthcare and limited availability of mental health options and providers. This presents a growing public health concern as the prevalence of mental health conditions has been increasing since the COVID-19 pandemic [3-4]. In both rural and non-rural communities alike, the prevalence of mental health conditions is on the rise.

The treatment and care of psychiatric illness is multifactorial involving a combination of therapeutic options that address patient symptoms both physiologically and psychologically. Such options include pharmacotherapy, psychotherapy, lifestyle modifications, and somatic treatments such as electroconvulsive therapy, deep brain stimulation, and transcranial magnetic stimulation [5]. Other somatic treatment options include massage therapy [6], exercise programs [7], and may even include osteopathic manipulative treatment (OMT) [8-11].

Previous studies on underserved populations in the US often exclude non-English speaking patients [12]. The majority of non-English speaking patients are Latino/Hispanic, who also represent the largest minority population in the US [13]. It has also been shown that the primary care setting is the most likely location for Hispanic women to seek treatment [14]. For mental health specifically, the depressive process of care and clinical outcomes have been shown to differ for the Latino and non-Latino populations [15]. As of 2017, Latinos made up 26-29% of the U.S. rural population [13].

Mental health disparity related to access is well documented, but little is known about patient attitudes toward and perceptions of their likelihood of receiving specific treatments after their diagnosis. Despite the existence of several treatment options, many patients still go untreated or do not have information about their availability and access. For example, most adults in the US who are diagnosed with depression do not receive treatment [16]. Thus, research that helps clinicians to better know and understand the factors that could improve patient adoption of a treatment modality with satisfying mental health outcomes in rural settings is imperative.

The overarching research questions underpinning this study are: To what extent is this patient population aware of the different mental health treatment options available? What are their perceptions, preference, and likelihood to seek treatment involving any of such options? What do they perceive as the barriers to seeking, access to, and use of mental health care in a rural setting? Hence, this study characterizes patient attitudes toward, beliefs regarding, and perceived barriers to treatment options for mental health conditions in a rural, underserved clinical setting. The study evaluates patients' attitudes toward different mental health treatment modalities, inclusive of OMT, to assess prior knowledge or familiarity with the technique.

## Materials and methods

Study design and population

A survey consisting of multiple choice and questions graded on a Likert scale was developed and administered to patients aged 18 years and older at the Campbell University Community Care Clinic (CUCCC) over the course of a four-month period in 2022. The objective was to assess patient attitudes and perceived barriers to mental health treatment. The CUCCC is a student-run free clinic for general healthcare that serves the uninsured and underserved in rural North Carolina including a large Spanish-speaking population. The clinic is held once each week. Participants were recruited at the time of check-in by being apprised of survey details and obtaining informed consent. The survey was offered in both English and Spanish languages to capture as many participants as could be representative of the target population. Patients unable to read, write, or provide full consent to filling out the survey were excluded. This study was approved under the Exempt category by the Campbell University Institutional Review Board (Number 717).

Survey questionnaire

Participants were provided with a paper survey composed of questions in the following three categories:

· Participant demographics: Patient demographic data on the variables age, gender, or race/ethnicity were obtained, and any patterned differences in the outcome responses were explored.

· Participant perceptions and likelihood of seeking behavioral (mental) health treatment (Tables 1-3): Asks patients to indicate whether they have received behavioral health treatment in the past. These data aim to identify the likelihood of seeking out specific treatment options as well as personal beliefs about behavioral health treatment and personally identified logistical barriers to receiving treatment [8,11,17-21].

**Table 1:** Table included in the survey assessing participants' likelihood of seeking specific treatment options. Responses were graded on a Likert scale.

If you had mental health concerns how likely would you be to seek out the following treatments? Please rate the following statements:
Take a prescription medication
Meet with a trained therapist in any capacity: virtual, in person, or in a group therapy session
Massage therapy, acupuncture, yoga
Seek guidance from a spiritual/church leader or get involved in a church community
Electroconvulsive therapy
Transcranial magnetic stimulation
Chiropractic treatment
Osteopathic manipulative treatment
Modifying exercise and eating habits
I would not seek treatment for a behavioral health/mental health concern.

**Table 2:** Table included in the survey assessing participants' feelings toward mental health treatment options. Responses were graded on a Likert scale.

The following statements ask about your feelings about mental health treatment options. Please rate the following statements:
I believe that mental health therapists actually care about their patients
I would be uncomfortable in a group therapy setting
Mental health treatment will make me crazy
I am afraid people would think less of me if I were being treated for mental illness
Having a mental illness would mean I am weak
Treatment for mental illness will make my symptoms better
I should be able to handle mental health problems on my own
I have gotten treatment for my mental health before and it did not help
Seeking mental health treatment takes too much time
I am too busy to seek additional treatment for my mental health

**Table 3:** Table included in the survey assessing logistical barriers to receiving treatment. Responses were graded on a Likert scale.

The following statements are about barriers to getting medical care. Please rate your agreement with the following statements:
I cannot attend appointments outside of normal clinic hours
I can only attend appointments on weekends
I do not have a reliable form of transportation
I cannot afford to pay for additional treatment

· Participant perceptions and knowledge of OMT (Table 4): Assesses patient familiarity with OMT, previous experiences with OMT, and the likelihood of having a physician perform manual treatments with their hands [8,11,21].

**Table 4:** Table included in the survey assessing participants; perceptions and knowledge of osteopathic manipulative treatment. Responses were graded on a Likert scale.

How likely would you be to have a physician perform the following with their hands:
Move your joints with stretching, gentle pressure, and force that causes direct relaxation to restore function
Use techniques similar to what massage therapists do to restore body function and joint motion
Help your body get back to its natural position, to help the body self-regulate and heal itself
Treat all muscles in the body, not just the areas causing the pain, to allow the body to work as a unit

Statistical analysis

Data were analyzed and are reported as percentages for survey responses of categorical variables. No inferential statistical analyses were conducted for these data, and no statistical significance was determined.

## Results

Demographics

**Figure 1:**
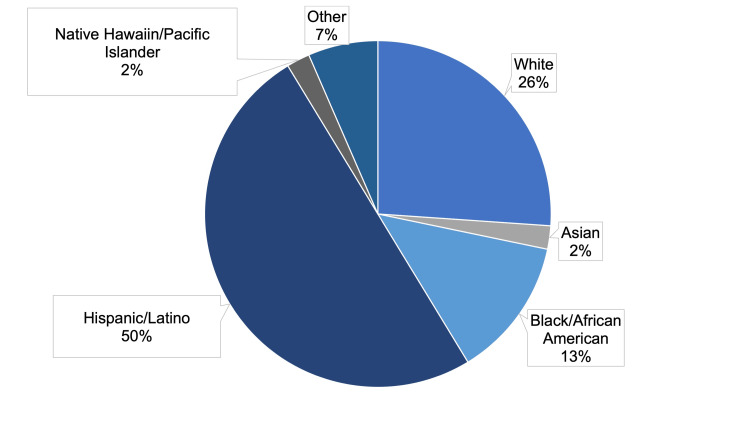
Survey respondent demographics with regard to respondents’ indicated race/ethnicity. %: percentage of respondents

A total of 46 responses were received out of around 105 administered, with a response rate of approximately 43.8%; 25 were received in English and 21 in Spanish. there were 20 male respondents and 24 female respondents, and two respondents preferred not to indicate their gender (Table 5). Concerning race/ethnicity, 26.1% of respondents were White, 2.2% were Asian, 13% were African American, 50% were Hispanic/Latino, 2.2% were Native Hawaiian/Pacific Islander, and 4.3% belonged to other category (Figure 1).

**Table 5:** Respondent demographics with regard to gender and language in which the survey was completed.

	Male	Female	Preferred not to say	Total
English	10	14	1	25
Spanish	10	10	1	21
Total	20	24	2	46

Mental health treatment options

**Figure 2:**
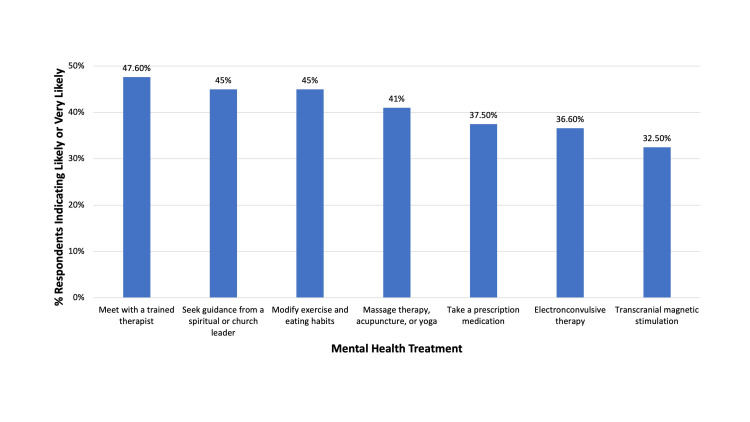
Positive survey responses to mental health treatment options in descending order of popularity. %: percentage of respondents

Among the respondents, 21.7% indicated that they had received some form of mental health treatment in the past. Meeting with a trained therapist in any capacity was listed as the most likely treatment to be sought if a respondent had mental health concerns, with 47.6% of respondents indicating they were either likely or very likely to choose it. Seeking guidance from a spiritual or church leader (45%) and modifying exercise and eating habits (45%) were the next most likely treatment to be sought, followed by massage therapy, acupuncture, or yoga (41%), or taking a prescription medication (37.5%) (Figure 2). Electroconvulsive therapy and transcranial magnetic stimulation were indicated as the two treatments most unlikely to be sought (36.6% and 32.5%, respectively) as well as the treatments that respondents were most unsure about (34.1% and 42.5%, respectively). While 20.5% of respondents indicated they would be likely or very likely to seek any treatment for mental health concerns, 51.3% of respondents indicated that they would be unlikely or very unlikely, 10.3% were neutral, and 17.9% were unsure.

Regarding feelings about mental health treatment providers, 66.7% of respondents believed that mental health therapists care about their patients. Of the respondents, 46.1% who reported receiving treatment for mental health in the past believed that it helped them. More than half (52.7%) of the respondents believed that mental health treatment would make their symptoms better.

Barriers to medical care

The most frequently indicated barrier to medical care by both Spanish- and English-speaking respondents was identified as cost, with 61% of respondents indicating that they cannot afford to pay for additional treatment. Clinic hours (21.4%), clinic location (19.6%), transportation issues (14.7%), and childcare problems (15%) were also indicated as barriers to care.

Osteopathic manipulative treatment knowledge

**Figure 3:**
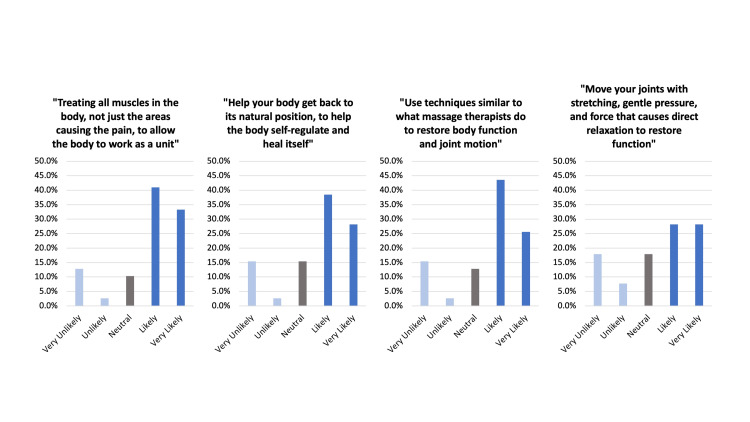
Survey responses to the question “How likely would you be to have a physician perform the following with their hands?” across all respondents who did not omit the questions. %: percentage of respondents

The majority (80.5%) of respondents indicated that they had never heard of OMT, with another 9.8% indicating they did not know what it was. All (100%) of the Spanish-speaking respondents reported unfamiliarity with OMT. Respondents were asked if they would be more likely than not to have a physician perform a technique on their body based on a set of defined OMT principles. Among those definitions “treating all muscles in the body, not just the areas causing the pain, to allow the body to work as a unit” was the most popular (74.3%). All other offered definitions were favorable: “use techniques similar to what massage therapists do to restore body function and joint motion” (69.2%), “help your body get back to its natural position to help the body self-regulate and heal itself” (66.7%), “move your joints with stretching, gentle pressure, and force that causes direct relaxation to restore function” (56.4%) (Figure 3).

## Discussion

We found that meeting with a trained therapist was the most likely form of mental health treatment that respondents would choose and that cost was the most indicated barrier to care. Additionally, it was observed that many of the respondents were unfamiliar with OMT, with all suggested definitions being favorable among respondents. Screening patients' knowledge may be necessary to inform best practices for effective mental health treatment [14]. Findings from this survey can help to mitigate adverse thoughts on seeking care for mental health treatment. Providers should better understand the role that stigma plays in patients’ willingness to engage in treatment [14].

Multiple factors such as those involving time limitations and financial constraints play into patient adherence and access to medical treatment. Often, types and characteristics of treatment barriers are specific to the geographic, cultural, and socioeconomic contexts of that patient population. This would suggest that barriers in rural areas differ compared to urban locales. This study found that cost was the most popular barrier to care among respondents. With the CUCCC serving a rural population, we may have expected a larger agreement with transportation issues due to geography. However, the CUCCC is also located rurally and can provide transportation to patients, thus making access easier, and this may be the reason the surveyed population did not indicate transportation as a larger barrier. Stigma toward mental health and psychiatric illness is another factor considered as one of the barriers to treatment [22]. Many solutions to improve healthcare delivery are currently being researched, with one example being telemedicine services that are increasing in popularity; however, telemedicine itself is not without its own barriers to adoption [23].

Many patients are familiar with the most common treatment options for mental health conditions such as talk therapy and medication. However, only 54% of patients responding to this survey showed improvement in response to pharmaceuticals and only 62% showed improvement after receiving psychotherapy [16]. This leaves a significant portion of patients without symptom improvement, highlighting the need for further treatment options. Of note, no protected healthcare information was collected during this survey, limiting the knowledge of patient's comorbidities. Patients presented to the CUCCC for various reasons, and as a general clinic, any problems could be seen, mental health or not.

An overwhelming majority of respondents had never heard of OMT. Notably, none of the Spanish-speaking respondents indicated familiarity with OMT. Different descriptions of OMT were provided in the survey, and the description of OMT referring to holistic medicine with the body working as a unit was the definition that made patients more likely to be favorable to OMT. Some research has suggested that OMT shows potential to be effective in helping the somatic symptoms of chronic mental health conditions [8-11]. Previous studies have shown positive outcomes in the use of OMT in the treatment of conditions commonly comorbid with psychiatric disorders including migraines [24], chronic pain, and chronic fatigue syndrome [25]. Osteopathic techniques heavily rely on touch [26]. Physical touch plays an important role in social bonding [27] and is known to improve coping with psychological distress [28-29].

One strength of this study was that it included a large percentage of Spanish-speaking respondents, which is, therefore, more representative of the student-run clinic population. The limitations associated with this study are the response rate and the resulting small sample size of respondents. This limited sample size, however, could be mostly attributed to the student-run clinic schedule of only meeting once per week. In addition, the education level of respondents was not assessed, and this could have added complexity to responses. There was difficulty with execution of such a survey at a student-run clinic, with procedural shortcomings such as lack of staff and prolonged appointment times with varying wait times.

The knowledge and understanding of patients’ perceived attitudes and barriers toward mental health care can provide insight to clinicians to improve patient outcomes and guide efforts in overcoming barriers to increase and expand mental health treatment availability and utilization by patients. With more available treatment options, there is potential for an increase in patient knowledge, understanding, and willingness to seek and be open to receiving such treatment options when necessary.

## Conclusions

Members of a student-run free clinic, both English- and Spanish-speaking, were surveyed about mental health treatment options, barriers to care, and familiarity with OMT. Meeting with a trained therapist was the most favorable form of mental health treatment indicated, cost was the most commonly indicated barrier to care, the majority of respondents were unfamiliar with OMT, and all suggested definitions of OMT were considered favorable by respondents. These responses can help guide clinicians in their efforts to overcome barriers to care for mental health illnesses and provide insight into potential treatment options for a rural and underserved population. Patient acceptance of and participation in mental health treatment is crucial for success, and understanding patient preferences can lead to a more effective treatment plan.
